# Hearables: feasibility of recording cardiac rhythms from single in-ear locations

**DOI:** 10.1098/rsos.221620

**Published:** 2024-01-03

**Authors:** Metin Yarici, Wilhelm Von Rosenberg, Ghena Hammour, Harry Davies, Pierluigi Amadori, Nico Ling, Yiannis Demiris, Danilo P. Mandic

**Affiliations:** Department of Electrical and Electronic Engineering, Imperial College London, London SW7 2AZ, UK

**Keywords:** wearables, hearables, electrocardiography, cardiac rhythm, electrocardiographic modelling, ear electrocardiography

## Abstract

The ear is well positioned to accommodate both brain and vital signs monitoring, via so-called hearable devices. Consequently, ear-based electroencephalography has recently garnered great interest. However, despite the considerable potential of hearable based cardiac monitoring, the biophysics and characteristic cardiac rhythm of ear-based electrocardiography (ECG) are not yet well understood. To this end, we map the cardiac potential on the ear through volume conductor modelling and measurements on multiple subjects. In addition, in order to demonstrate real-world feasibility of in-ear ECG, measurements are conducted throughout a long-time simulated driving task. As a means of evaluation, the correspondence between the cardiac rhythms obtained via the ear-based and standard Lead I measurements, with respect to the shape and timing of the cardiac rhythm, is verified through three measures of similarity: the Pearson correlation, and measures of amplitude and timing deviations. A high correspondence between the cardiac rhythms obtained via the ear-based and Lead I measurements is rigorously confirmed through agreement between simulation and measurement, while the real-world feasibility was conclusively demonstrated through efficacious cardiac rhythm monitoring during prolonged driving. This work opens new avenues for seamless, hearable-based cardiac monitoring that extends beyond heart rate detection to offer cardiac rhythm examination in the community.

## 1. Introduction

The use of wearable technologies for monitoring vital signs has become increasingly widespread in society, for both recreational and medical purposes. Most often, these devices are integrated into wearable garments and accessories, and are concealed and miniaturized for convenience of the user. The most common choices for wearable vital signs monitoring technologies are smart watches, which typically use the photoplethysmogram (PPG) to provide continuous monitoring of pulse and respiration, and chest straps, which may also record the electrocardiogram (ECG), in addition to the PPG. However, PPGs lack the information necessary to understand the functioning of the heart [1], while chest-worn devices are not suitable for everyday use due to their obtrusive nature. Consequently, alternative solutions that provide ECG measurements in a convenient and user-friendly manner have attracted significant research interest.

One such solution is the ‘hearable’ device—a wearable that fits in the ear and can serve both as an audio-accessory and a platform for health monitoring [2,3]. For devices worn on the head and around the ear region, such as hearables, the stability of the head relative to the brain and other vital organs during everyday activities, such as walking, sleeping and sitting, results in superior brain and vital signs monitoring capability relative to devices worn on the limbs, such as smart watches/rings [4]. Consequently, hearable devices that are worn on the ear, in addition to those devices worn on the regions of skin surface surrounding the ear on the scalp and neck, are gaining popularity. For example, Da He *et al.* [5] and Casson and co-workers [6] demonstrated the efficacy of behind-the-ear and temporal-scalp-based recordings of ECG, ballistocardiogram (BCG) and PPG for heart rate monitoring, while Celik and co-workers [7,8] provided evidence of similar results when measurements were taken from combined on-ear and neck configurations. Despite the success of these proof-of-concept studies, the difference in ECG potential from the various ear and lateral-head locations is not yet well understood.

Previous work by Von Rosenberg *et al.* [9] and Chanwimalueang *et al.* [10] investigated the ECG potential over various regions of the head surface and focused on ECG recording positions embedded within the interior surface of a ‘smart helmet’. Each channel under consideration consisted of a bi-lateral pairing of electrodes, whereby electrodes were positioned symmetrically about the midline sagittal plane on both the left and right sides of the head. The performance of each channel under consideration was evaluated with respect to the accuracy of an *R*-peak detection algorithm [10]. Results from the study demonstrated that head ECG channels comprising electrode positions in closer proximity to the heart were most reliable. In addition, Guler *et al.* [11] systematically analysed electrode positioning for bi-lateral pairings of behind-the-ear electrodes and identified the mastoid area behind the helix to be most suitable for cardiac monitoring. Through experimentation alone, however, it is difficult to rigorously evaluate the efficacy of an ECG channel, since the effect of the manifold factors that can influence the ECG signal is not clear [12]. For example, muscle activity, brain activity and variations in skin–electrode contact can all contribute to the ECG signal [13,14]. Consequently, the current experimental studies are limited by either a lack of empirical evidence or support from theoretical models.

A comprehensive theoretical approach to mapping ECG potentials on the head can be achieved through forward modelling [15,16]. This process involves applying Maxwell’s equations to a dielectric model of the body in order to estimate the propagation of cardiac potentials from the heart to the head. This offers the advantage of examining the ECG potential in isolation; in the absence of brain and muscle activity, and ECG hardware related artefacts. Furthermore, such modelling can enable the evaluation of multiple wearable ECG channels simultaneously, in a time- and cost-efficient manner. Early ECG modelling was based on simplified representations of the human body (for example, a homogeneous conductive sphere [17]); however, more advanced, anatomically accurate models have since been produced which include separate shells representing the heart and surrounding body [18], and realistically shaped geometries for specific organs in the chest cavity [19–22]. A model which enables the simulation of ECG propagation to the head and ear locations was first introduced by Von Rosenberg *et al.* [12]. A whole-body model incorporating tissue from the torso and head was employed to provide a rigorous theoretical basis for the investigation of the feasibility of high-quality head ECG. In addition to simulations, an analysis of the ECG surface potential from head and in-ear channels was conducted. Moreover, the characteristic timing and shape of the ECG from the different channels was evaluated through both measurements and simulations, opening new avenues for wearable head-based cardiac monitoring, beyond heart rate detection.

Ideally, ear-ECG measurements should be conducted from a device worn on a single ear; however, the ECG potential available on the single ear, or the surrounding locations on the scalp and neck, is not well understood. To fill this void, this work sets out to combine forward modelling and the experimental approach outlined in [12], in order to assess the ECG potential on a single side of the head, ear and neck. Since the standard cardiac rhythm obtained via the Lead I ECG on the chest is established within the medical setting, and is commonly measured by single-lead ECG wearables [23,24], we further determine the similarity between the cardiac rhythms obtained via the scalp, ear and neck-ECG measurements and the Lead I ECG measurement. After the single ear-ECG potentials are established and understood, we proceed to demonstrate the real-world feasibility of ear-ECG monitoring throughout prolonged simulated driving.

## 2. Methods

Within this study, the feasibility of recording cardiac rhythms from single-ear ear-ECG channels is evaluated through both experimentation and ECG forward modelling. Two experiments, termed Experiment A and Experiment B, were conducted in order to (i) map the ear-ECG potential over the neck, ear and scalp regions, and subsequently (ii) demonstrate the feasibility of ear-ECG measurement under challenging real-world recording conditions. In addition, through forward simulations in a whole-body ECG volume conductor model, the biophysics principles behind the ear-ECG measurements were established.

### 2.1. Experimental design

#### 2.1.1. Experiment A: ECG mapping on the single ear

Previous studies have investigated the feasibility of wearable ECG monitoring via ear, temporal scalp and neck based sensors [5–8,11]; however, the characteristics of the ECG signal at each of these locations have not yet been the subject of comprehensive examination. The aim of Experiment A was to establish the difference in the ECG cardiac rhythm between the ear, scalp and neck locations on a single side of the head, through measurements on subjects at rest. Recordings were conducted for 10 min for each participant, as this duration enabled evaluation of ear-ECG extraction for measurement periods suited to wearable cardiac monitoring, e.g. multiple seconds/minutes. GRASS Ag/AgCl cup electrodes were used for all recordings. ECG was recorded from three channels of interest on (i) left lateral scalp, (ii) left ear and (iii) left lateral neck, and a reference channel located on the wrists. Electrode configurations for each ECG channel employed within Experiment A are displayed in table 1 and are as follows. In order to record the ECG from the position of the left ear, the electrodes were positioned on the left concha and the left helix. In order to record the ECG from the position of the left scalp, a lower scalp electrode was positioned at a distance of *L*/2 above the left helix electrode, while an upper scalp electrode was placed at a distance of *L* above the lower scalp electrode, where *L* equals the separation between the left helix electrode and left concha electrode. An equivalent positioning system was employed for lower and upper neck electrodes, whereby the neck electrodes were positioned below the left concha electrode (table 1). A standard limb-ECG channel was also created between the left and right wrist, whereby electrodes were positioned on the left and right volar central zones (below the palm). It was previously demonstrated that this channel produces a cardiac rhythm in high correspondence with the standard Lead I [12,25]. The electrode positioning for the ear, scalp and neck-ECG channels was devised such that each channel spanned an equal distance across the head surface and that the three channels were vertically aligned. Prior to the application of the electrodes, the skin at each location was prepared through cleaning with medical wipes and abrasion with NuPrep gel. A layer of 10–20 conductive paste was also applied to the electrodes prior to placement, in order to improve conduction between the skin surface and the electrodes. Measurements were conducted via a custom bio-amplifier programmed to digitize the potential difference between the upper and lower scalp electrodes (scalp-ECG), the helix and concha electrodes (single-ear ear-ECG), the upper and lower scalp electrodes (neck-ECG) and the left and right wrist electrodes (wrist-ECG). A sampling rate of 500 Hz was used and the helix electrode served as a ground. During the measurements, subjects were seated and instructed to close their eyes while minimizing eye movements, and movement of the head, neck and arms. The recordings were performed under Imperial College London ethics committee approval JRCO 20IC6414, and full informed consent was provided by each subject.

**Table 1.** Experiment A electrode positioning. Bipolar channels were created between pairs of electrodes on the scalp, ear and neck, on the left side of the head. For the scalp and neck channels, the vertical positioning of each electrode is shown within parentheses. The vertical length *L* is equal to the vertical separation between the positioning of the helix and concha electrodes. In addition to the ear and head ECG channels, a high signal-to-noise-ratio, reference ECG was recorded between the wrists.

ECG channel	electrode	
	positive terminal	negative terminal
wrist	left volar wrist	right volar wrist
scalp	lower scalp (left helix + *L*/2)	upper scalp (left helix + 3*L*/2)
ear	left concha	left helix
neck	lower neck (left concha − 3*L*/2)	upper neck (left concha − *L*/2)

#### 2.1.2. Experiment B: real-world feasibility

To demonstrate real-world feasibility of single-ear ear-ECG, 1 h long measurements were conducted throughout a simulated driving task, during which a prolonged recording period, and driving-related head and body movement, and jaw clenching presented real-world recording challenges. Five subjects who also participated in Experiment A were recruited for Experiment B. Each subject was instructed to drive for a period of 1 h in a driving simulator while maintaining a central road position and a constant, moderate speed. Participants were seated in a driving rig (steering wheel and accelerator pedal), while wearing a FOVE-0 VR headset which displayed the driver-view of the car and the driving course [26]. The driving environment was built in Unreal Engine 5 [27]. Measurements of ear- and wrist-ECG were conducted continuously throughout the 1 h trial. In total, measurements of three ear-ECG channels were conducted: left single ear, right single ear and cross-ear. Although cross-ear ECG was not the focus of this study, since the feasibility of cross-ear recordings under more challenging recording scenarios had not yet been established, the cross-ear channel was included in the recording configuration for Experiment B (recordings on subjects at rest were reported in [12,28]). Following the same electrode positioning convention as in Experiment A, for the two single ear-ECG channels that were located on the left and right ears in Experiment B, electrodes were positioned on the helix and the concha. For the ground, the electrode was positioned on the ipsilateral ear lobe. With regard to the cross-ear ECG channel, the electrodes were positioned on the left helix and right concha, and the left ear lobe for grounding. For the reference ECG, electrodes were positioned on the left and right volar regions of the wrists. All electrode configurations employed in Experiment B are listed in table 2. With regard to sensor material, since the objective of Experiment B was to establish the feasibility of real-world measurements of single ear-ECG, recordings of the ECG from the concha were performed with wearable, flexible electrodes, presented in [29]. For the helix and ear lobe positions, as a result of the relatively large and flat surface area available for electrode placement, standard GRASS Ag/AgCl cup electrodes were used. The wearable electrodes employed within this study for concha recordings were constructed from a fabric of silver-coated thread that is interwoven with elastic fibres. In [2], for five subjects, ear electroencephalography (EEG) measurements collected via electrodes of this type were shown to provide stable electrode impedance for the duration of a normal working day. During the recording period, everyday activities such as walking, eating and talking were unrestrained. In the present study, the electrodes were positioned on the surface of malleable non-allergenic silicone, which were moulded to the surface of each individual’s concha. This method of in-ear-ECG acquisition was both time and cost effective since the silicone substrates are inexpensive and readily available, and also straightforward to use. In order to improve the skin–electrode connection, prior to application of both the wearable and Ag/AgCl electrodes, the skin surface was cleaned with medical wipes and lightly abraded with NuPrep gel. Subsequently, a thin layer of Signa Gel conductive gel was applied to the surface of the wearable electrode. For the Ag/AgCl electrodes, 10–20 conductive paste was applied. All of the electrophysiological channels described above were connected to a g.tec g.USBamp (2011) bio-amplifier and were sampled at a rate of 1.2 kHz.

**Table 2.** Experiment B electrode positioning. Bipolar channels were created between pairs of locations on the left ear, right ear and between the ears. Additionally, a high signal-to-noise-ratio, reference ECG was recorded between the wrists.

ECG channel	electrode		
	positive terminal	negative terminal	ground
wrist	left volar region	right volar region	left ear lobe
left ear	left concha	left helix	left ear lobe
right ear	right helix	right concha	right ear lobe
cross-ear	left helix	right concha	left ear lobe

Within this study, while cardiac rhythm extraction was evaluated from data recorded throughout the entire 1 h recording, examples of cardiac rhythms that were recorded during the final 10 min of the driving task were plotted for visualization. Examples of such data were provided for the purpose of conveying cardiac rhythm extraction during real-world scenarios, since, after 50 min of driving, it is likely that the wearable sensors would have been subject to multiple periods of motion and dislodging that are common to real-world measurements.

### 2.2. Signal processing

The ECG data from Experiments A and B were processed in the same manner, with the exception of a down-sampling procedure which was employed for data from Experiment B, in order to reduce the sampling frequency to 300 Hz. All signal processing was performed in MatLab 2022a (MathWorks Inc., MA, USA). During pre-processing, the head-/ear-ECG data were bandpass-filtered between 0.5 Hz and 30 Hz using a third-order Butterworth filter. For the reference data, a third-order Butterworth bandpass filter (0.5–95 Hz) and a second-order IIR notch filter with a centre frequency of *f*_*c*_ = 50 Hz and a bandwidth of *w* = 5 Hz were employed. Following filtering, the extraction of median cardiac rhythms was performed. First, the timings of cardiac rhythms from within the recordings were obtained via the Pan-Tompkins algorithm on the reference ECG data [30,31]. Subsequently, 600 ms windows around each *R*-peak (ranging from 200 ms prior to and 400 ms post the timing of the *R*-peak) were extracted from the scalp, ear, neck and reference ECG data. On average, over the course of the 10 min recordings, 690 ± 160 (mean ± standard deviation) cardiac rhythms were detected for each participant. Next, median cardiac rhythms were obtained for varying ensemble sizes, *N* = 2, 8, 16, 32, 64, 120, 180, 240, 300, 420, 540, where the ensemble size, *N*, refers to the number of consecutive cardiac rhythms which were used during the calculation of the median. For all selected values of *N*, the maximum number of median cardiac rhythms were obtained from the data available. For example, for a measurement segment containing 100 consecutive cardiac rhythms, for an ensemble size of *N* = 2, 100 − *N* = 98 median cardiac rhythms were calculated. In this way, cardiac rhythm monitoring was evaluated for data from the entire trial. The cardiac rhythm evaluation procedure consisted of calculating four performance metrics based on the similarity between median cardiac rhythms from a given channel and the grand-median cardiac rhythm from the Lead I equivalent reference ECG channel, where the grand-median refers to the median of the maximum ensemble size available for that subject. Finally, the inter-subject average of the metrics for each channel was calculated. Algorithm 1 describes the signal processing pipeline for the cardiac rhythm extraction and subsequent performance evaluation, while the formulae for the calculation of the performance metrics are given below:

(i) The *Pearson correlation coefficient* between the cardiac rhythms from a given channel of interest and the grand-median from the reference channel:
  2.1 $$r=\frac{\sum (x_{i}-\bar{x})(y_{i}-\bar{y})}{\sqrt{\sum {\left(x_{i}-\bar{x}\right)}^{2}{\left(y_{i}-\bar{y}\right)}^{2}}},$$
  where *r* denotes the correlation coefficient, *x*_*i*_ are the values of the head/ear cardiac rhythm, $\bar{x}$ is the mean of the values of the head/ear cardiac rhythm, *y*_*i*_ are the values of the reference cardiac rhythm and $\bar{y}$ is the mean of the values of the reference cardiac rhythm. The value for the Pearson correlation coefficient for optimal ECG channel performance is equal to 1.
(ii) The *wave amplitude ratio*. For cardiac rhythms from a given head/ear channel, the ratios between the amplitude of the *R*-wave and the *P*-, *Q*-, *S*- and *T*-waves normalized with respect to corresponding ratios for the grand-median of the reference channel as
  2.2 $$\mathrm{war}=\frac{1}{4}\sqrt{\sum {\left(\frac{a_{\;j}b_{r}}{b_{\;j}a_{r}}\right)}^{2}},$$
  where war denotes the wave amplitude ratio, *a*_*j*_ the amplitudes of the *P*-, *Q*-, *S*- and *T*-waves of the head/ear cardiac rhythm, *a*_*r*_ the amplitude of the *R*-wave of the head/ear cardiac rhythm, *b*_*j*_ the amplitudes of the *P*-, *Q*-, *S*- and *T*-waves of the reference cardiac rhythm, and *b*_*r*_ the amplitude of the *R*-wave of the reference cardiac rhythm. The value for wave amplitude ratio at optimal ECG channel performance is equal to 1.
(iii) The *wave timing error*. For cardiac rhythms from a given head/ear channel, the difference between the timings of the *P*-, *Q*-, *S*- and *T*-waves relative to the *R*-wave, and the corresponding relative timings obtained from the grand-median of the reference channel as
  2.3 $$\delta_{\mathrm{wt}}=1/4\sqrt{\sum {\left((c_{\;j}-c_{r})-(d_{\;j}-d_{r})\right)}^{2}},$$
  where *δ*_wt_ denotes the wave timing error, *c*_*j*_ the timings of the *P*-, *Q*-, *S*- and *T*-waves of the head/ear cardiac rhythm, *a*_*r*_ the timing of the *R*-wave of the head/ear cardiac rhythm, *b*_*j*_ the timings of the *P*-, *Q*-, *S*- and *T*-waves of the reference cardiac rhythm, and *b*_*r*_ the timing of the *R*-wave of the reference cardiac rhythm. The value for wave timing error at optimal ECG channel performance is equal to 0.
(iv) The *normalized variance*. For a given head/ear channel, the root-mean-square error (RMSE) between the grand-median cardiac rhythm and all individual cardiac rhythms from the same channel is given by
  2.4 $$\delta_{\mathrm{nv}}=\frac{1}{\sigma_{y}}\frac{1}{N}\sqrt{\sum_{n}^{N}{\left(x_{i}-y_{i}\right)}^{2}},$$
  where *δ*_nv_ denotes the normalized variance, *x*_*i*_ are the values of the head/ear cardiac rhythm, *y*_*i*_ are the values of the grand-median head/ear cardiac rhythm and *σ*_*y*_ is the standard deviation of the grand-median head/ear cardiac rhythm. The value for normalized variance at optimal ECG channel performance is equal to 0.

In order to calculate performance metrics (ii) and (iii), the amplitude and timing of the *P*-, *Q*-, *R*-, *S*- and *T*-waves from each of the computed median cardiac rhythms were extracted. The code for the algorithm which extracts the timing of the *P*-, *Q*-, *R*-, *S*- and *T*-wave peaks from windowed ECG data is provided online (see the data accessibility statement), while a description of the algorithm steps is provided in the electronic supplementary material.

**Algorithm 1.** Signal processing steps.

1 Bandpass-filter the head-/ear-ECG signals using a third-order Butterworth filter with a lower cut-off frequency of $f_{\min}=0.5\;\mathrm{Hz}$ and an upper cut-off frequency of $f_{\max}=30\;\mathrm{Hz}$, to give filtered_ECG.

2 Bandpass- and notch-filter the reference channel, respectively, using a third-order Butterworth filter with a lower cut-off frequency of $f_{\min}=1\;\mathrm{Hz}$ and an upper cut-off frequency of $f_{\max}=95\;\mathrm{Hz}$, and a second-order IIR filter with a centre frequency of $f_{c}=50\;\mathrm{Hz}$ and a bandwidth of w=5 Hz, to give filtered_reference.

3 Perform R-peak detection via the Pan-Tompkins algorithm on filtered_reference.

4 Extract cardiac rhythms from filtered_ECG around the identified R-peak timings obtained in Step 3.

5 Find the median cardiac rhythms for the ensemble sizes: *N* = 2, 8, 16, 32, 64, 120, 180, 240, 300, 420, 540.

6 Calculate four metrics for the assessment of the median cardiac rhythms from individual channels, for the various ensemble sizes: (i) Pearson correlation between median cardiac rhythms in a given channel and the grand-median from the reference channel (1), (ii) wave amplitude error between median cardiac rhythms in a given channel and the reference channel (2), (iii) wave timing error between median cardiac rhythms in a given channel and the reference channel (3) and (iv) normalized variance in the channels—the RMSE of the differences between the individual cardiac rhythms and the grand-median cardiac rhythm, divided by the standard deviation of the grand-median cardiac rhythm of the channel under consideration (4).

### 2.3. ECG modelling

#### 2.3.1. Volume conductor geometry

**Figure 1.**
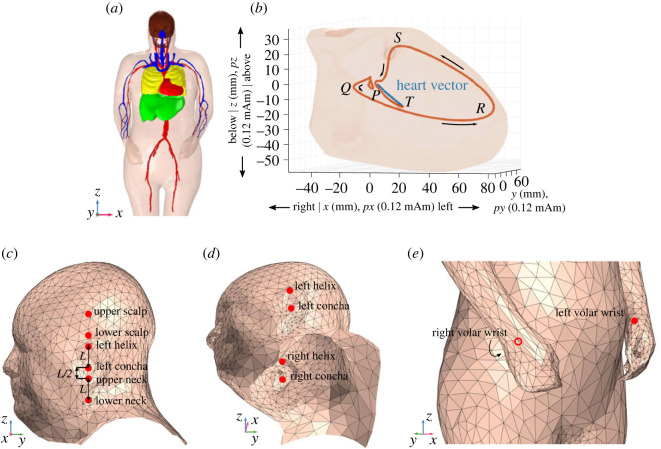
ECG volume conductor modelling. (*a*) Three-dimensional model structure: organs and tissue in the torso and head enclosed within the skin surface. (*b*) Heart vector: the orientation and magnitude of the current dipole *p* in *px*, *py* and *pz* directions; the heart vector at one point in time is shown in blue, and the trace of the tip of the heart vector from the start of the cycle until the current position (axes in 0.12 mA m) is shown in orange; the heart muscle is shown in pink in the background (axes in millimetres). The direction of heart vector change is indicated with arrows. (*c*) Head-/ear-ECG electrode positioning in Experiment A shown on the model: potential differences between electrodes on the upper and lower scalp (scalp-ECG), helix and concha (ear-ECG) and upper and lower neck (neck-ECG) were extracted. Measurements *L* and *L*/2 which were used during electrode positioning are also shown. (*d*) Ear-ECG electrode positioning in Experiment B: potential differences between electrodes on the left helix and left concha (left ear-ECG), right helix and right concha (right ear-ECG) and left helix and right concha (cross-ear-ECG) were extracted. (*e*) Reference wrist-ECG electrode positioning in Experiments A and B: the electrode positioning on the left and right volar region of the wrists (palm-side) is shown on the model.

The geometry within the presented model is based on the VHP-Female Computational Phantom v. 2.1 and v. 2.2 [32]—a dataset consisting of three-dimensional geometry for major organs and tissues in the human body. The phantom was edited in instances where computational problems were encountered with the mesh of the structures in the model in the modelling software (COMSOL Multiphysics [33]). The model comprised geometric representations for major structures surrounding the heart and in the head, and a complete whole-body volume enveloped by an outer skin structure; the top-half of the model is shown in figure 1*a*. For the purpose of realistic modelling of electric fields within the body, a large sphere of radius *r* = 3.3 m, filled with air, surrounded the body and provided an electrical ground in the model. The complete mesh consisted of 560 630 domain elements and 72 286 boundary elements, and the average edge length was 6 mm.

#### 2.3.2. Electric properties of the body and cardiac current sources

The relevant electric properties of the modelled human tissues (conductivity and relative permittivity at an excitation frequency of 15 Hz) were extracted from the database in [34], and are provided in the electronic supplementary material (table SM1). Previous research has revealed that isotropic and anisotropic treatments of the heart structure in simulations of ECG yield similar results [35]. Therefore, an isotropic treatment of the heart structure was adopted within the present model. Multi-source modelling of the cardiac potential has been shown to perform best for prediction of the ECG potential on the torso [36]; however, owing to the relatively large distance between the head and heart, the present model employs single source modelling. The heart source dynamics over the course of a single cardiac cycle are shown in figure 1*b*; a comprehensive description of the electrical dynamics of the cardiac cycle is provided in [37]. The source dipole in the presented model is a superposition of three orthogonal vectors representing the projection of the cardiac potential over the course of a single cardiac cycle. The three orthogonal directions that are used to describe the heart vector are the left to right, front to back and below to above directions (respectively shown as the *x*-, *y*- and *z*-axis in figure 1*b*). The heart vectors were acquired from real-world measurement from a subject with no known cardiac abnormalities. The cardiac cycle was taken to start and end 200 ms before and 400 ms after the maximum of the *R*-wave, at a sampling frequency of 500 Hz.

#### 2.3.3. Simulated ECG

In order to establish the biophysics principles behind the ECG channels of interest within Experiments A and B, forward predictions of cardiac potential at ECG channel positions on the body were extracted from the model. Specifically, the cardiac potential was extracted from positions listed in tables 1 and 2 (also displayed on the model in figure 1*c*–*e*). According to the bipolar electrode pairings in tables 1 and 2, differential potentials were then calculated. In this way, simulated cardiac rhythms based on volume conductor physics provided a benchmark for real-world ECG measurement. Performance metrics (1)–(3) were also calculated for the simulated cardiac rhythms. In addition, to provide a visualization of the ECG potential over the skin surface of the neck, scalp and ears, forward predictions of the surface potential over these areas at the time of the *R*-peak are provided.

## 3. Results

### 3.1. Correspondence between simulation and measurement: Experiment A

**Figure 2.**
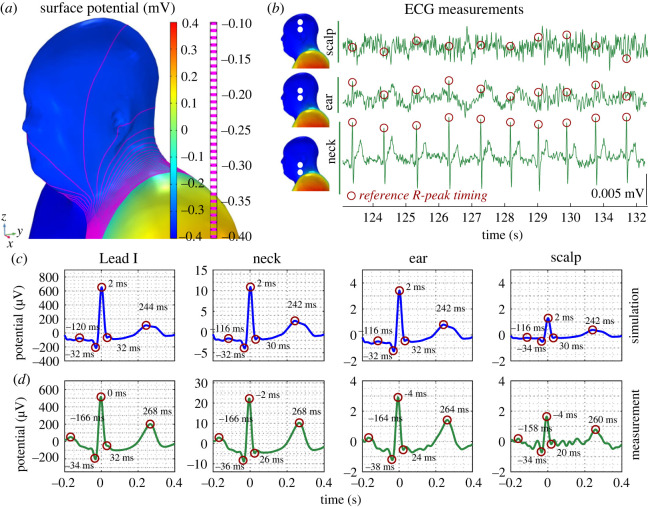
Simulated and recorded cardiac electric potentials. (*a*) Simulated electric potential on the head surface and upper torso at the time of the *R*-wave peak (in millivolts) in the biophysics model. (*b*) Recorded ECG traces from the scalp-, ear- and neck-ECG channels from a single subject. The ECG potentials at the instance of the *R*-peaks in the reference (Lead I) channel are indicated (red circles). (*c*) Simulated cardiac rhythms in scalp-, ear- and neck-ECG based on volume conduction. (*d*) Measurements of cardiac rhythms in scalp-, ear- and neck-ECG. Results are based on averaging over a 10 min recording and exhibit clear cardiac rhythms in correspondence with the simulated predictions in (*c*). For both (*c*) and (*d*), ECG potentials at the timings of the *P*-, *Q*-, *R*-, *S*- and *T*-waves (from left to right) are circled. The timing of each eave is indicated.

In order to provide rigorous theoretical support to the ear-ECG measurements, forward predictions of ECG volume conduction were conducted. Specifically, the ECG potential at the timing of the *R*-peak on the left side of the head and upper torso was investigated and is shown in figure 2*a*. Note the large potential difference between the left and right sides of the torso; on positions where conventional Lead I electrodes are placed. By contrast, observe the reduction in potential difference across the surface of the head (where the ear and head ECG channels are based) relative to the neck and torso. A decrease in potential difference from the neck position to the ear and then to the scalp is clearly demonstrated by the equipotential lines (magenta) spanning the range −0.1 mV to 0.4 mV. This stems from the narrow neck structure which impedes ECG potential as it propagates from the heart to the head surface.

Next, ECG traces obtained from the scalp-, ear- and neck-ECG channels of Subject 6 were plotted (figure 2*b*) and the expected positionings of the *R*-peaks were highlighted across all channels using the timings obtained from the reference ECG data via the Pan-Tompkins algorithm. Observe that the *P*-, *Q*-, *R*-, *S*- and *T*-waves are identifiable in the neck-ECG channel, while in the ear-ECG trace, only *R*-peaks are intermittently identifiable as a result of the noisy ECG signal. For the scalp trace, waves from the ECG cardiac rhythms are not visible. These results further demonstrate the impedance of the ECG potential by the narrow structure of the neck. In figure 2*c*,*d*, respectively, cardiac rhythms obtained via simulation are displayed alongside grand-median cardiac rhythms obtained through measurement (*N* = 607 cardiac rhythms), with annotated timing of the *P*-, *Q*-, *R*-, *S*- and *T*-waves. Simulated cardiac rhythms from the neck-, ear- and scalp-ECG were found to be in good correspondence with the Lead I ECG cardiac rhythm, with respect to timing and shape of the multiple cardiac rhythm waves, as shown in figure 2*c*. The cardiac potential projection plane that is shared by these channels explains the observed similarity in the simulated cardiac rhythms. Furthermore, the amplitude of the simulated cardiac rhythms was once more observed to decrease from the neck to the ear, and from the ear to the scalp. The correspondence between the neck-, ear- and scalp-ECG cardiac rhythms and the Lead I cardiac rhythm was also evident in the measurements, as shown in figure 2*d*. Moreover, the aforementioned decrease in amplitude between the neck-, ear- and scalp-ECG channels was also observed.

### 3.2. Ear-ECG cardiac rhythms: Experiment A

**Figure 3.**
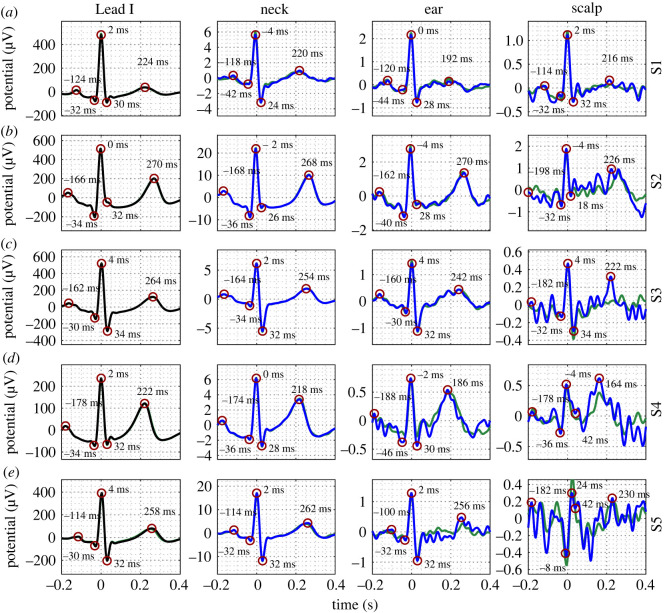
Cardiac rhythms from wrist-, scalp-, ear- and neck-ECG of five subjects (*a*–*e*). Reference Lead I cardiac rhythms are displayed along the first column. ECG acquired after *N* = 240 rhythms (blue) and *N* = 540 rhythms (green). ECG potentials at timings of *P*-, *Q*-, *R*-, *S*- and *T*-waves in each channel are circled for the *N* = 240 cycle ECG. Timings of the waves are indicated next to each wave.

Examples of median cardiac rhythms obtained from the first *N* = 240 and *N* = 540 cardiac rhythms for Subjects 1–5 are displayed in figure 3. Data are displayed for the Lead I equivalent wrist-, scalp-, ear- and neck-ECG. The timings of the cardiac rhythm waves are also annotated. The *N* = 240 cardiac rhythms obtained from all subjects via the neck-ECG were observed to consistently exhibit correspondence with the Lead I cardiac rhythm, as shown in figure 3*a*–*e*. Note the similarity in the timing and shape of the waves in the cardiac rhythms. Visual inspection of the cardiac rhythms from the neck for the *N* = 240 and *N* = 540 ensembles reveals that the SNR of the cardiac rhythms were equivalent. For the *N* = 240 cardiac rhythms obtained via the ear-ECG, data from seven out of the ten subjects (Subjects 1–3 and 7–10) exhibited correspondence with the Lead I cardiac rhythm. Examples of such data for Subjects 1–3 are provided in figure 3*a*–*c*. For three out of ten subjects (Subjects 4–6), the correspondence with the Lead I was less evident. Examples of such results, for Subjects 4 and 5, are displayed in figure 3*d*,*e*. The cardiac rhythm from the ear for the *N* = 240 and *N* = 540 ensembles were visually equivalent for Subjects 1–3, 5–8 and 10; however, for Subjects 4 and 9, increases in SNR were observed. With regard to the scalp-ECG, for the *N* = 240 cardiac rhythms, as evidenced in figure 3*a*,*e*, correspondence with the Lead I wave morphology was not observed. The cardiac rhythms from the scalp for the *N* = 240 and *N* = 540 ensembles were visually equivalent. For completeness, examples of median cardiac rhythms obtained from the first *N* = 240 and *N* = 540 cardiac rhythms for Subjects 6–10 are provided within the electronic supplementary material.

**Figure 4.**
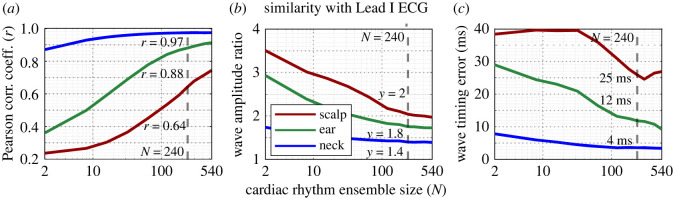
Performance metrics for median scalp-, ear- and neck-ECG cardiac rhythms for varying ensemble sizes (number of consecutive cardiac rhythms used during the calculation of the median), *N*. (*a*) Pearson correlation coefficient of the cardiac rhythms from a given channel with the grand-median cardiac rhythm from the reference channel. (*b*) Wave amplitude ratio between the *R*-peak and *P*-, *Q*-, *S*- and *T*-peaks from the cardiac rhythms from a given channel, normalized by equivalent ratios from the grand-median cardiac rhythm from the reference channel. (*c*) Wave timing error for the *P*-, *Q*-, *S*- and *T*-waves relative to those obtained from the grand-median cardiac rhythm from the reference channel. A vertical dashed line indicates the values at *N* = 240 rhythms.

Performance metrics which evaluated the quality of cardiac rhythms extracted from scalp-, ear- and neck-ECG channels were calculated at various cardiac rhythm ensemble sizes, ranging from *N* = 2 to *N* = 540, and are displayed in figure 4. Each performance metric was evaluated with respect to the similarity between the cardiac rhythms obtained from the channel under consideration and the grand-median cardiac rhythm from a Lead I equivalent reference channel. Values for performance metrics at a cardiac rhythm ensemble size of *N* = 240 for each channel are annotated on the plots. The neck-ECG channel was observed to perform the best with respect to the Pearson correlation, wave amplitude ratio and wave timing error, as displayed in figure 4*a*–*c*, respectively. Favourable performance metric values at relatively low ensemble sizes demonstrate such superior performance. Values of *r* = 0.87, war = 1.7 and *δ*_wt_ = 8 ms, were observed for *N* = 2, while values of *r* = 0.97, war = 1.4 and *δ*_wt_ = 4 ms were observed for *N* = 240. The values for all three performance metrics of the ear-ECG channel were observed to approach those of the neck-ECG channel for higher values of *N*; values of *r* = 0.88, war = 1.8 and *δ*_wt_ = 12 ms were observed for *N* = 240. However, at lower ensemble sizes, the performance was severely decreased; values of *r* = 0.36, war = 2.9 and *δ*_wt_ = 29 ms were observed for *N* = 2. The performance of the scalp-ECG channel was less favourable at low ensemble sizes. However, although the performance improved with higher values of *N*, still a smaller increase in performance was observed; values of *r* = 0.64, war = 2 and *δ*_wt_ = 38 ms were observed for *N* = 240.

### 3.3. Correspondence between simulation and measurement: Experiment B

**Figure 5.**
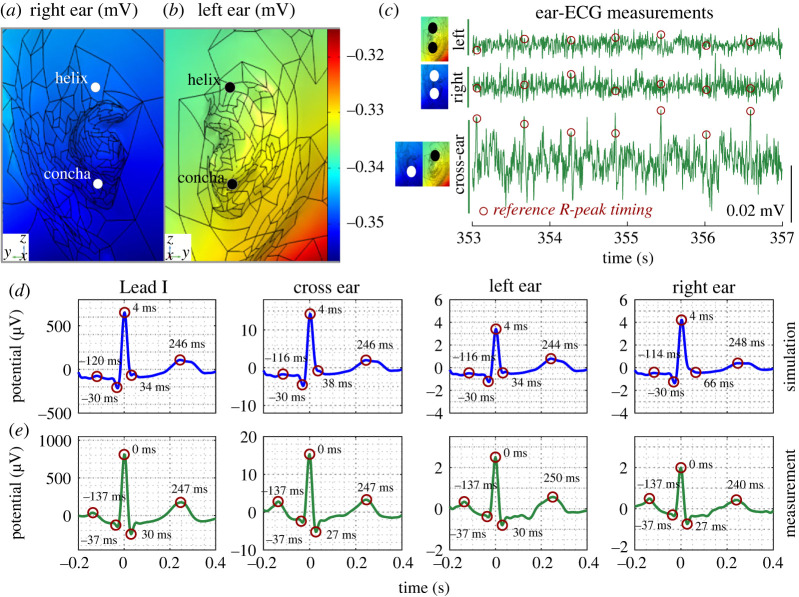
Simulated and recorded cardiac electric potentials. Simulations of (*a*) left ear and (*b*) right ear surfaces on the model at the timing of the *R*-peak. The outline of the mesh of the model is shown in black to improve the clarity of the shape of the ear surface. (*c*) Recorded ECG traces from left ear, right ear and cross ear-ECG channels from a single subject. The ECG potentials in each channel at the timings of the *R*-peaks in the reference (Lead I) channel are circled. (*d*) Simulated and (*e*) measured cardiac rhythms in wrist, left ear, right ear and cross ear-ECG. (*e*) Cardiac rhythms based on averaging over a 60 min recording. ECG potentials as the timings of the *P*-, *Q*-, *R*-, *S*- and *T*-waves (from left to right) are circled. The timing of each wave is indicated.

To support the ear-ECG channels under consideration in Experiment B, forward predictions of ECG volume conduction were first analysed. Specifically, the surface potential over the left and right ears at the timing of the *R*-peak during the simulated cardiac cycle is visualized in figure 5*a*,*b*. The positions of the helix and concha are also indicated. The potential difference between the helix and concha positions on a single ear was approximately 0.005 mV, while the potential difference between electrode positions on opposing ears was approximately 0.04 mV. This difference arises as a result of the smaller distance between electrodes on a single ear, relative to the distance which separates electrodes positioned on the left and right ears. In figure 5*c*, examples of ECG traces obtained from the left-, right- and cross-ear ECG channels of Subject 4 are displayed and the expected positioning of the *R*-peaks are highlighted across all channels, using the timings obtained from the reference ECG data via the Pan-Tompkins algorithm. Note that only the *R*-waves are identifiable in the cross-ear ECG channel, while in the left and right ear-ECG traces, waves from the ECG cardiac rhythms are not visible as a result of the higher noise levels. In figure 5*d*,*e*, respectively, cardiac rhythms obtained via simulation and grand-median cardiac rhythms obtained through measurement during the driving task (*N* = 4086 cardiac rhythms) are displayed, with annotated timing of the *P*-, *Q*-, *R*-, *S*- and *T*-waves. In figure 5*d*, simulated cardiac rhythms from the left-, right- and cross-ear ECG were observed to be in good correspondence with the Lead I ECG cardiac rhythm, with respect to timing and shape of the multiple cardiac rhythm waves. In figure 5*e*, the correspondence between the left-, right- and cross-ear ECG cardiac rhythms and the Lead I cardiac rhythm was also evident in measurements. In addition, the lower potential difference over the surface of a single ear relative to the surface of opposing ears was also observed in ECG measurement.

### 3.4. Ear-ECG cardiac rhythms: Experiment B

**Figure 6.**
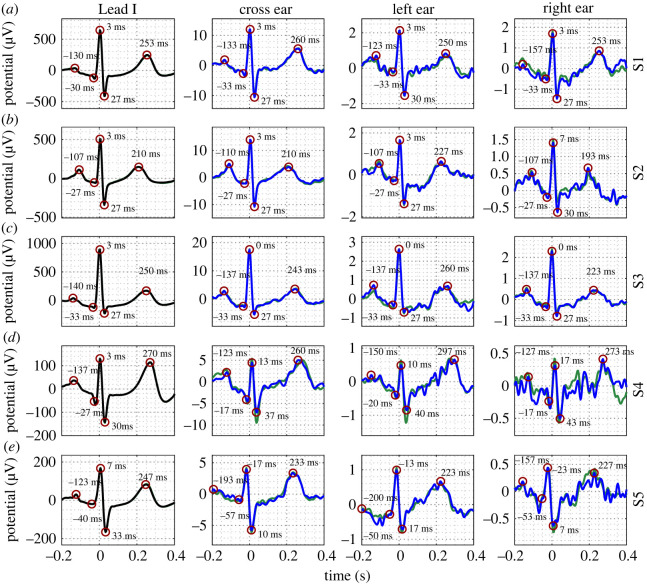
Cardiac rhythms from wrist, left ear, right ear and cross ear-ECG of five subjects (*a*–*e*). Reference Lead I cardiac rhythms are displayed along the first column. The ECG acquired after *N* = 240 rhythms (blue) and *N* = 600 rhythms (green). The ECG potentials at timings of *P*-, *Q*-, *R*-, *S*- and *T*-waves in each channel are circled for the *N* = 240 cycle ECG. Timings of the waves are indicated.

Median cardiac rhythms extracted from ensembles of size *N* = 240 and *N* = 540 from the final 10 min of the driving task are displayed in figure 6. Data are displayed for the Lead I equivalent wrist-, left-, right- and cross-ear ECG measurements. The timings of the cardiac rhythm waves are also annotated. For the *N* = 240 median cardiac rhythms obtained via the cross-ear ECG, correspondence with the Lead I cardiac rhythm was observed. The *N* = 240 and *N* = 540 cardiac rhythms from the cross-ear ECG were visually equivalent. For the *N* = 240 cardiac rhythms obtained via the left ear-ECG, data from Subjects 1–3 exhibited correspondence with the Lead I cardiac rhythm (figure 3*a*–*c*). For Subjects 4 and 5, however, correspondence with the Lead I was less evident. For example, the *T*-wave of the cardiac rhythm from Subject 4 (figure 3*d*) exhibited more noise than the equivalent wave from the Lead I cardiac rhythm, while the *P*-wave of the cardiac rhythm from Subject 5 (figure 3*e*) exhibited a different morphology. The cardiac rhythms from the left ear-ECG channel for the *N* = 240 and *N* = 540 ensembles were visually equivalent for Subjects 1–3; however, for Subjects 4 and 5, increases in SNR were observed. For the *N* = 240 cardiac rhythms obtained via the right ear-ECG, data from Subjects 1 and 3 exhibited correspondence with the Lead I cardiac rhythm (figure 3*a*,*c*), while for Subject 2, the correspondence with the Lead I was less evident. On the other hand, for Subjects 4 and 5, correspondence with the Lead I was not evident. The cardiac rhythms from the right ear-ECG channel for the *N* = 240 and *N* = 540 ensembles were visually equivalent for all subjects.

**Figure 7.**
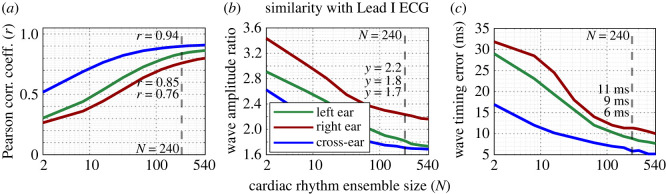
Performance metrics for median left-, right- and cross-ear ECG cardiac rhythms for varying values of cardiac rhythm ensemble size (number of consecutive cardiac rhythms used during the calculation of the median), *N*. (*a*) Correlation of the cardiac rhythms with the grand-median Lead I cardiac rhythm, (*b*) RMS amplitude ratio between the *R*-peak and *P*-, *Q*-, *S*- and *T*-peaks for a given channel, normalized by the values from Lead I and (*c*) RMSE of the timings of the *P*-, *Q*-, *S*- and *T*-waves relative to the *R*-wave between a given channel and the Lead I channel. A vertical dashed line indicates the values at *N* = 240, which are also displayed in table 3.

Performance metrics which evaluated the quality of cardiac rhythms extracted over the course of the entire driving trial from left-, right- and cross-ear ECG channels were also calculated at multiple cardiac rhythm ensemble sizes, ranging from *N* = 2 to *N* = 540, and are displayed in figure 7. The values for performance metrics at a cardiac rhythm ensemble size of *N* = 240 for each channel are highlighted. The cross-ear ECG channel was observed to perform the best with respect to the Pearson correlation, wave amplitude ratio and wave timing error, as displayed in figure 7*a*–*c*, respectively. This is evidenced through the more favourable performance metric values at relatively low ensemble sizes. Note that, for the cross-ear ECG channel, values of *r* = 0.71, war = 2.2 and *δ*_wt_ = 12 ms were observed for *N* = 10, whereas, for the left and right ear-ECG channel, respectively, values of *r* = 0.5, war = 2.6 and *δ*_wt_ = 22 ms and *r* = 0.4, war = 2.9 and *δ*_wt_ = 27 ms were observed for *N* = 10. However, at a value of *N* = 240, the left and right ear-ECG channel performance metrics were observed to approach the favourable values of the cross-ear ECG channel. It was also observed that, for all three performance metrics, the left ear-ECG channel performed marginally better than the right ear-ECG channel. Performance metric values from each channel from Experiments A and B for an ensemble size of *N* = 240 are displayed alongside performance metrics based on the simulated cardiac rhythms from each channel under investigation in both Experiments A and B in table 3.

**Table 3.** Mean performance metrics for *N* = 240 cardiac rhythms for scalp-, cross ear-, left ear-, right ear- and neck-ECG channels. (i) Pearson correlation coefficient, (ii) wave amplitude ratio, (iii) wave timing error and (iv) normalized variance. Results under the column heading ‘meas.’ were calculated using measured cardiac rhythms, whereas results under the column heading ‘sim.’ correspond to results for the simulated cardiac rhythms. Values for left ear-ECG were calculated for data collected during both Experiments A and B, values for right ear- and cross ear-ECG were calculated with data from Experiment B, and values for scalp- and neck-ECG were calculated with data from Experiment A.

	(i) Pearson corr. coeff.		(ii) wave amplitude ratio		(iii) wave timing error		(iv) normalized variance
ECG channel	meas.	sim.	meas.	sim.	meas. (ms)	sim. (ms)	meas.
(Exp. A) wrist	1	1	1	1	0	0	1
(Exp. A) neck	0.97	0.99	1.4	1	4	1	1
(Exp. B) cross-ear	0.94	0.99	1.7	1.1	6	2	3
(Exp. B) left ear	0.85	0.99	1.8	1.1	9	2	4
(Exp. A) left ear	0.88	0.99	1.8	1.1	12	2	6
(Exp. B) right ear	0.76	0.99	2.2	1.2	11	2	9
(Exp. A) scalp	0.64	0.97	2	1.5	25	3	12

## 4. Discussion

### 4.1. Experiment A: ECG mapping on the single ear

In §3.1, it was established that the cardiac rhythms obtained via the scalp-, ear- and neck-ECG channels were in good correspondence with the cardiac rhythm obtained via the Lead I ECG channel. Importantly, predictions of cardiac rhythms from scalp-, ear-, neck- and Lead I ECG based on volume conduction were observed to match real-world measurements. Thus, the correspondence between the cardiac rhythm from the scalp-, ear- and neck-ECG channels and the cardiac rhythm from the Lead I ECG channel was comprehensively demonstrated. The implication of such correspondence is that the wearable ECG channels under consideration could provide diagnostic value in the same manner as the Lead I ECG channel.

In §3.2, the cardiac rhythms obtained via a moderate measurement length of the scalp-, ear- and neck-ECG (*N* = 240) were visualized, and the performances of the channels with increased and decreased measurement length were evaluated. The performance of the scalp-ECG channel during real-world measurements was shown to be considerably worse than that of the ear- and neck-ECG channels. This primarily stems from (i) poorer electrode–skin contact through hair on the scalp, (ii) lower amplitude ECG potential at the location of the scalp relative to the ear and neck and (iii) large amplitude background noise (EEG and temporal muscle-EMG) at the location of the temporal scalp. This was further emphasized through the values for normalized variance (table 3). The ear-ECG performance was shown be favourable for *N* = 240, evidenced through the Pearson correlation coefficient equal to 0.88, and the wave timing error equal to 12 ms. These results indicate that, in short-time, real-world scenarios, the single-ear ear-ECG could be used in order to record cardiac rhythms with *shape* and *timing* that are equivalent to the Lead I ECG. However, a wave amplitude ratio equal to 1.8 implies that the *amplitude* of the single ear-ECG may not be equivalent to that of the Lead I ECG. It is likely that the discrepancy between the amplitude information in the cardiac rhythms from the head-based ECG and the Lead I ECG is caused by the averaging procedure, which smooths the cardiac rhythms. Therefore, a more sophisticated cardiac rhythm extraction method could be used to improve the amplitude profile of the in-ear-ECG, thus potentially improving diagnostic value. With regard to the neck-ECG, it was observed in figure 2*b* that cardiac rhythms similar to the Lead I can be obtained without averaging. The performance metrics for the Pearson correlation coefficient and the wave timing error, for a value of *N* = 2, are demonstrative of this, and conclusively demonstrate the feasibility of high-quality neck-ECG.

### 4.2. Experiment B: real-world feasibility

In Experiment B, the focus was on solely ear-based ECG monitoring (without the use of the surrounding neck or scalp surface), and the feasibility of real-world measurements was tested. Two more ear-ECG channels were also investigated: the right ear ECG and the cross-ear ECG. Therefore, prior to evaluation of the performance of the channels during the driving task, the ECG potential available to each of these channels was investigated through both modelling and analysis of grand-median measurements. Previous modelling and real-world measurement, by Von Rosenberg *et al.* [12], demonstrated the correspondence between the cardiac rhythms from the cross-ear ECG and the Lead I ECG, which was once more observed through both measured and simulated cardiac rhythms in §3.3. With regard to the right ear-ECG channel, measured and simulated cardiac rhythms also demonstrated a good correspondence with the cardiac rhythm obtained via the Lead I ECG channel. These results indicate that all three of the investigated in-ear-ECG channels could provide a Lead I equivalent ECG measurement.

In §3.4, cardiac rhythms obtained via a moderate measurement length of the left-, right- and cross-ear ECG (*N* = 240), were visualized, and the performances of the channels with increased and decreased measurement length were evaluated. The *N* = 240 cardiac rhythms from the cross-ear ECG exhibited high correspondence with the Lead I for all subjects at the end of the driving task, as shown in figure 6, demonstrating the real-world feasibility of such recordings. However, for the single ear-ECG, the correspondence with the Lead I for the *N* = 240 cardiac rhythms was less evident for subjects which exhibited lower Lead I ECG amplitude. For example, the Lead I *R*-peak amplitude was equal to 200 μV for Subject 4, in contrast to Lead I *R*-peak amplitudes greater than 400 μV for Subjects 1–3, while the respective left ear *R*-peak amplitudes were less than 1 μV and over 2 μV. As such, while evidence of real-world feasibility was provided for single ear-ECG during driving (for 3 out of 5 subjects), more sophisticated methods of cardiac rhythm extraction may be required in order to reliably extract the low amplitude single-ear cardiac rhythms for subjects who exhibit low amplitude standard ECG.

The performance of the left-, right- and cross-ear ECG channels was comprehensively evaluated over the course of 1 h recordings from five subjects. Values for the Pearson correlation coefficient, wave amplitude ratio and the wave timing error were all favourable for *N* = 240 cardiac rhythms from the cross-ear ECG channel, conclusively evidencing real world feasibility. Furthermore, correlation results for the cross-ear ECG in [12] (*r* = 0.96 for 4 min at rest) from a different cohort of 6 subjects are in good agreement with the results in this study (*r* = 0.94 for *N* = 240 or roughly 4 min, while driving). With regard to the left ear-ECG channel, the values for all performance metrics were marginally less favourable relative to the cross-ear ECG, and with respect to the right ear-ECG channel, the performance was further degraded. This decreased performance was most evident for lower ensemble sizes, suggesting that shorter-time applications of cardiac monitoring could be better suited to the cross-ear ECG relative to the single-ear ECG. Values for normalized variance (table 3) further indicate the superiority of the cross-ear ECG relative to the single-ear ECG. Nevertheless, the real-world feasibility of cardiac monitoring via single ear *N* = 240 cardiac rhythms was rigorously demonstrated through favourable scores for all three performance metrics, paving the way for ultra-wearable, single-ear, in-ear cardiac rhythm monitoring.

## 5. Limitations and future work

In [12], a collocated micro-electromechanical sensor placed beneath the surface of the ear-ECG fabric electrodes was used to detect pressure waves in the ear canal surface associated with the heart beating (i.e. the BCG). This served as a reference for the timing of the cardiac rhythms in the ear-ECG signal detected by the fabric electrodes and, in conjunction with a matched-filtering technique [9,10], enabled reliable identification of cardiac rhythm timing in the noisy ear-ECG signal. Such techniques were not the subject of the present study; therefore, timings of the *R*-peaks that were extracted from the reference ECG channels were used. However, for the purpose of further demonstrating real-world feasibility of the single ear-ECG, a study of such multi-modal single ear-ECG measurements, partnered with the development of more sophisticated machine learning approaches to both the localization and high SNR extraction of cardiac rhythms should also be investigated. Some progress in these areas has been achieved in recent work on deep-matched filtering for enhanced cardiac rhythm localization in cross-ear ECG [38].

In figure 6, it was observed that, for all subjects, the amplitudes of the cardiac rhythms from the right ear were lower than those from the left ear. This decreased amplitude could have given rise to the observed degraded performance metrics for the right ear-ECG relative to the left ear-ECG. On the other hand, it is also possible that poorer skin–electrode contact, and other experimental factors, could have adversely biased the results for the right ear-ECG, given that data from only five subjects were analysed. An alternative cause of the degraded performance of the right ear-ECG channel is that the Lead I cardiac rhythm was not a suitable benchmark for this channel. Indeed, while correlations based on simulations suggest that the right ear-ECG channel is suitably benchmarked against Lead I (*r* = 0.99), it is possible that the model suffers from inaccuracies for the right ear-ECG in particular. In consequence, future studies should address the correspondence of the ear-ECG leads with a wider set of standard ECG leads.

Our findings demonstrate the potential of ear-ECG for capturing cardiac rhythms with shapes that are very similar to Lead I from the standard limb leads, even during challenging, real-world recordings. This framework holds promise for examining heart conditions that are visible in multiple consecutive cardiac rhythms in Lead I, including but not limited to myocardial infarction (the ST segment is elevated), first-degree atrioventricular block (the PR interval is longer than 200 ms), atrial fibrillation (absence of the *P*-wave, found in 2–3% of the population in Europe and the USA [39–41]), sinus tachycardia (increased heart rate and a shortened *P*–*T* duration) and atrial flutter (an increased frequency of the *P*-wave relative to the QRS complex as a result of rapid atria contraction) [42–44]. Moreover, the proposed ear-ECG framework offers the possibility of 24/7 continuous and unobtrusive cardiac monitoring and can alert the user to the presence of universal signatures of heart malfunction, such as absent *P*-waves and elongated ST segments. Such a new perspective can be beneficial to many existing applications, such as remote monitoring for patients with cardiac conditions such as arrhythmias, or heart failure [45], sports and fitness monitoring of athletes [46] and assessment of the effect of physical strain and stress in workplace environments [47,48].

## 6. Conclusion

The ability to monitor ECG is envisaged to be a key feature in future wearable health systems. The ear is widely recognized as a primary position to record physiological signals from, as a result of its stable location relative to the vital organs during everyday activities and its ability to house commonplace accessories such as earbuds. However, the feasibility of recording the ECG signal that is available over the surface of a single ear had not yet been established, either theoretically or through experimentation. To this end, first, the difference in ECG potential on the ear and in surrounding regions of the neck and scalp has been investigated. These results have been shown to support both existing and prospective wearable ECG platforms that use scalp, ear and neck locations. Measurements of single ear-ECG on ten subjects, during an ideal scenario of resting while sitting, have demonstrated, for the first time, the characteristic timing and shape of the ECG signal available at the single ear location. Further measurements, including both single-ear and cross-ear ECG, on five subjects during a 1 h driving task have demonstrated real-world feasibility of ear-ECG. Both the cross-ear and single-ear ECG have been shown to be robust in real-world environments over prolonged recording periods, thus providing conclusive evidence for the use of such technology in society and in eHealth. Future work will consider the integration of additional cardiac monitoring sensors into the ear worn platform, such as the PPG and BCG, to aid the localization of *R*-peaks, together with the investigation of various cardiac conditions via the ear-ECG.

## Data Availability

Data and code are available from the Dryad Digital Repository: https://datadryad.org/stash/share/Jtyc03eWrU6VG0rg1QkEKSGhOm_ejGGYbRPsYvV65w4 [49]. Supplementary material is available online [50].
